# American Medical Association

**Published:** 1866-07

**Authors:** 


### PROCEEDINGS OF MEDICAL SOCIETIES.

### AMERICAN MEDICAL ASSOCIATION.

### *Section on Practical Medicine and Obstetrics.*

#### DISCUSSION ON DIPHTHERIA.

The section was organized by the appointment of Dr. Lake J. Tefft, of New York, Chairman, and Dr. W. B. Bibbins, of New York, Secretary.

Dr. H. D. Holton of Putney, Vermont, Chairman of the Committee on Diphtheria, as it had prevailed in the United States, made a report by reading an elaborate and interesting paper. He gave a history of the disease from its first appearance in this country, more than a century since. He insisted that diphtheria should not be confounded with follicular tonsilitis and kindred affections; in fact, nothing should be dignified with the name that did not present the characteristic exudation, with swelling of the cervical glands, etc. Neither should it be confounded with scarlatina or croup. Tables were presented showing the fatal month, the proportion of deaths at various ages of the three diseases. The disease was divided into diphtheria simplex, diphtheria maligna or gangrenosa, and tracheal diphtheria. He entered at some length into the discussion of the sequelæ, stating that they were paralysis, rheumatism, and general cachexia.

After discussing the various plans of treatment, he divided it into local and general. Such mild cathartics should be used as the case seemed to require, although nothing like active purgation should be indulged in. Chlorate of potassa should be given pulverized with an equal amount of sugar, a little placed in the mouth, and allowed to run down the throat, that the local as well as the constitutional effect might be obtained.

Sulph. quin. and tr. ferri chloridi should be used as occasion required; also alcoholic liquors, either alone or combined with chinchona bark; wine whey, milk punch, and beef-tea were to be used to support the patient when a nourishing diet could not be taken.

The local application should be such as would prevent the absorption of fluid portions of the exudation. For this purpose persulphatum ferri was recommended; also the following: ℞. Tr. ferri chloridi, chlorat. potassæ, *aa* ʒj; glycerine pure, ℥ ij. Misce. Either of these to be carefully applied with a soft brush. Externally, an infusion of hops in lye or wood ashes would be found best for any hot fomentation.

The following deductions were made: 1st. That diphtheria has occurred as an epidemic from time to time from the first settlement of this country; 2d. That it is a distinct disease, on no account to be confounded with scarlatina or croup; 3d. That it is particularly a disease of childhood, although it exempts no age; 4th. That it is communicable to that degree that it is the duty of every physician to separate the infected from those that are well, particularly in children; 5th. That it is a disease primarily affecting the blood, consequently the treatment to be effective must be not local but general, and of such a nature as to eradicate or neutralize the poison; such local treatment should, however, be used as will prevent the absorption of fluid portions of the exudation; 6th. That when the exudation has invaded the trachea, the only hope of saving the patient is in tracheotomy.

On motion of Dr. Ellsworth Eliot, of N. Y., the report was referred to the Committee on Publication.

Dr. King wished to take exception to the remark made in this paper that when the membrane is thrown off from the trachea the patient will almost always get well. He had seen two cases, one a man and the other a child, in which the membrane had been thrown off and both had died. He could not believe that the cast-off membranes were ever reabsorbed. He stated that he had ceased to use local applications for anything more than their antiseptic effects; and for this purpose he thought that the solution of common salt was as good as anything else.

Dr. Gallagher, of Pittsburgh, Pa., expressed himself much pleased with the report. He regretted, however, that the author had made no reference to the use of alum as a local application. He had used that remedy with the happiest effects, and had more confidence in it than all the others. He had used also with great satisfaction the ordinary "hard cider," with a view of assisting the elimination of the morbid materials by the kidneys.

Dr. Worthington Hooker, of New Haven, stated that there was a great tendency in the profession to run into extremes in reference to treatment. He was not disposed to think that any disease could be successfully treated by following any one plan to the exclusion of others. The really rational plan was to follow all the indications in the particular case as they might arise. There were many marked distinctions between croup and diphtheria, but he chose only to refer to the want of that severe constitutional disturbance in the former which is never absent in the latter disease.

Dr. McCollum, of Vermont, stated that he had seen 200

cases of the disease in his county within the last five years. He had seen three or four cases recover after the membrane had been cast off. He never used gargles except as disinfectants. As regards ice, he had noticed benefit from its use in a great many cases. He had also been in the habit of allowing his patients to inhale the vapor of acetic acid, and with marked benefit.

Dr. Gross, of Pittsburgh, stated that he had had a large experience in the treatment of diphtheria in his district, and had come to the conclusion that the best means of treatment consisted, first in the use of calomel as an antiphlogistic, followed by the very free use of chlorate of potash. He had seen 400 or 500 cases of the disease since he instituted this plan of treatment, but had not met with a single fatal case. He also, in conjunction with the foregoing remedies, made use of the solution of nitrate of silver (20 grains to the ounce) as a local application, as well as saturated solutions of alum and lemon-juice on crushed ice.

Dr. Bibbins, of N. Y., stated that some time since the question was asked him by Prof. A. Clark—What was the number of cases of diphtheria which he had met with in dispensary practice? Dr. Bibbins, before he was prepared to answer such a question, wished to know from Dr. Clark what he understood to be true diphtheria—whether the essential symptoms of the disease were considered to be adenitis, false membrane, fever, and depression of the vital powers. "Of course," replied Dr. Clark, "Dr. A. Jacobi, who had studied this subject very thoroughly, has published the fact that these symptoms are always essential to the disease." A certain proportion of cases must be distributed to each individual; and such being the fact, they could not but believe that when gentlemen report such a large number of cases as occurring in their practice they do not take into account the necessary symptoms of diphtheria; that a great many report cases which they call diphtheria, which other practitioners are not willing to admit as such. He remarked that the average mortality of true cases of diphtheria among dispensary patients was thirty-three per cent., and did not suppose that under any treatment this mortality could be much decreased. Dr. A. Clark had reported the same rate of mortality. Dr. Bibbins was led to believe, from the cases which he had met with, that the tonic and supporting plan of treatment was the only reliable one.

Dr. Shumway, of N. Y., had used with great benefit, as local applications, the nitrate of silver stick, the chlorate of potash, and the chlorate of ammonia. He apprehended that the great difficulty with many patients that were convalescent was not that they did not get medicine enough, but that there was paralysis of the throat which prevented them from swallowing them. In those cases he had used nutritive injections into the stomach with very good results.

Dr. Davis, of Ill., spoke in this connexion of blood-poisoning in many diseases, diphtheria among the rest. He thought that there was yet a great deal to be learned with reference to the true pathology of these so-called blood diseases. The query had arisen with him whether diphtheria really depended primarily upon a poison in the blood, or whether the altered condition of the blood itself, instead of being primary, was itself a secondary consequence of a prior change in the properties of the organized tissues of the human body. He discussed this question at considerable length, showing the probability that there might be such a fundamental error in nutrition, as that assimilation and disassimilation was so interfered with that the blood was poisoned in consequence.

Dr. Hewitt remarked that he had great faith in the solvent power of both alum and chlorate of potash on the diphtheritic membrane. He had taken pieces of the membrane that had been separated, and had dissolved them entirely in solutions of these salts. His treatment was to take a glass tube, pass it back into the fauces, and inject one or other of the solutions directly against the affected parts.

Dr. McIntyre, of Concord, N. H., was of the opinion that if there was any error in nutrition, it was secondary to the formation of the membrane in the throat, which, by obstruction to the respiration, interfered with the proper oxidation of the blood. This he considered to be the starting-point of the constitutional trouble. For the purpose of aiding the system in the elimination of the poison in the later stages of the disease he had used colchicum seed, guaiac, and nitrate of potash, with much satisfaction.

Dr. Taylor, of Iowa, did not think that there was any good to be attained by suggesting this and that remedy for a disease which had existed in every section of the country; for different remedies must be used in different localities, and he thought the best guide for any general plan of treatment was to be found in a proper, thorough, and enlightened view of the pathology of the disease.

### INHALATION OF NEBULIZED FLUIDS.

Dr. J. Solis Cohen, of Philadelphia, read a paper entitled "Remarks on the Inhalation of Nebulized Liquids, and their Topical Application to Inflamed and Ulcerated Surfaces."

The reader stated that his design was not so much to present a review of the knowledge of the profession on these topics, or to bring forward any of his peculiar views, as much as to give a resume of the results thus far obtained from recent experience, with a view of eliciting upon a subsequent occasion a valuable paper on the whole subject of the Therapeutics of Inhalation, which should embody the experience of a number of observers, and furnish the profession with authoritative data for their general investigation, inasmuch as the literature on this subject, especially in this country, was very meagre and unsatisfactory.

The paper stated that the idea of treating diseases of the respiratory organs by inhalation, which should bring remedies directly in contact with diseased surfaces, was as old as the healing art itself; for what was more natural than to imitate the entrance of the vivifying breath of life in its access to the economy? The records of classic Greece and Rome show that this principle was taken advantage of in the inhalation of volatile substances, and in the residence of patients upon the seashore, and in the neighborhood of volcanoes. This method of treatment fell into disuse, and was again resorted to many times, and had fallen into permanent neglect for a long period, until the discoveries of oxygen leading to further chemical research, and the promulgation of the exhilarating effects following inhalation of the nitrous oxide gas, again directed attention to the subject of inhalation as a remedial agent. Then the ammoniacal exhalations from cattle yards, stables, etc., became largely employed in many affections, until too extended empirical application to all the ills of life meeting with failure, led the whole system into disuse, even for these affections for which it was originally intended. The discoveries of the properties of chlorine, iodine, etc., of the effects of the smoking of opium among the Chinese, led to the employment of similar substances; and the whole series of narcotics, resins, and all substances producing vapor became employed in this manner.

Observations of the diseases affecting workers in minerals, metals, etc., and the post-mortem revelations of the scalpel, proving the entrance of particles of dust into the lungs, led to the therapeutic employment of powders by inhalation.

After some other general remarks of a similar nature, the paper went on to relate that within a few years a new method of presenting *liquid substances* for inhalation *as such*, had become much employed in Europe, and seemed to promise an extensive field of success in the treatment of the whole class of pectoral troubles.

Every one is familiar with the effect of a stream of water directed upon the side of a house, how a large portion of the volume of water is directed back in a shower of small streams, or coarse spray. In certain bathing establishments in Germany, many liquid applications are made on this principle to the surface of the body in the treatment of affections of special organs, and of the general system likewise. Streams of the medicated liquid are forced against metallic plates on the walls of the bathing apartments, and fall over the desired surfaces in this shower or coarse spray.

It having been noticed that amelioration of concomitant chest affections seemed to follow this exposure for other purposes, patients were sent to these establishments to respire the air thus impregnated. The beneficial results obtained at the Inhalatorium of Dr. Auphan, of Eaux-les-Bains, established in 1849, led to similar establishments elsewhere, until finally, Sales-Girons, who, in connexion with Flube, had in 1856 erected an inhalatorium at Pierre-fonds, conceived the idea of constructing on this principle a portable apparatus which could be employed by patients at their residences, and thus admit of much wider application. Sales-Girons exhibited an instrument of this kind to the Academy in 1858. His apparatus consisted of a condensing syringe, which forced a very fine stream of liquid against a convex metallic button, converting it in deflection into a very finely divided spray, having much the appearance of a dense fog or mist, the separate particles of which, suspended as they are for a time in the atmosphere, playing around much like the dust observed in a sunbeam, can be drawn into the lungs by inspiration.

The paper referred to several of these apparatus, especially the modification of the tubes of Bergson, and the employment of steam by Siegle, in lieu of the compressed air, and stated that experiments upon living animals proved the entrance of these particles into the air-cells; though for various reasons, the spray striking against many surfaces during its ingress, its dissipation into a much larger volume than could be received into the mouth, etc., but a small quantity of the liquid thus nebulized (the writer thought not more than twelve per cent.) could reach the smaller bronchial ramifications.

The writer thought that the application in this manner of liquid substances of known strength directly to the diseased surfaces would prove more prompt in effect, whether for good or ill, than when the entire current of the circulation became the vehicle of communication, perhaps affecting the general system when not desirable. The mucous membrane of the lungs is quick to absorb, secretes no chemical product to modify chemical medicines, and has no temporary ingesta to interfere with the direct action of remedial agents applied to it.

Although the results thus far obtained are far from being positive, and many of them are unsatisfactory and negative, still they are such as to justify careful research into this new method of medication.

Some conclusions were then given as the results of recorded experience; the results of the writer's personal experience and observations, and the results of extensive experiments, carefully conducted by Dr. J. M. Da Costa, at the Pennsylvania Hospital, as well as those obtained by the latter named gentleman in his private practice.

A few remarks followed as to the manner of preparing medicines for employment in this way, and their application when different parts of the air-passages were to be medicated.

Allusion was made to the employment of this system of nebulization for local applications to inflamed and ulcerated surfaces on the exterior of the body, and a certain distance within the various cavities, and an instrument devised for this purpose was exhibited, to demonstrate that in a second or two a layer of a caustic or other solution, many times more delicate than any film which should be deposited by the slightest touch of a hair pencil, could be directed upon an ulcerated or inflamed surface, whether of large or small surface. A continuous application of course can be kept up when desired, and in this connexion the method was recommended for the production of local anæsthesia for minor operations in surgery—a subject at present exciting a good deal of attention, and brought to the notice of the profession by Dr. B. W. Richardson, of London.

On motion, the paper was referred to the Committee on Publication, for publication in the Transactions of the year; but at the request of the writer, the motion was modified to call for the appointment of a special committee to report on the "Therapeutics of Inhalation" at the next annual meeting of the Association.

On motion, the paper was received.

Dr. J. E. Clawson, of Delaware, moved that the Section appoint a Special Committee on the Therapeutics of Inhalation, to consist of three members, and that the paper of Dr. J. S. Cohen, just read, be referred to that Committee. Carried.

The Chairman appointed as such Committee:

Dr. J. Solis Cohen, Philadelphia; Dr. J. M. DaCosta, Philadelphia; Dr. L. Elsberg, New York.

Adjourned.

### *Section on Medical Jurisprudence, Physiology and Hygiene.*

### FIRST SESSION, MAY 1, 1866.

The session was organized at Concordia Hall, 3 P. M., May 1, by electing Dr. Wilson Jewell, of Pennsylvania, Chairman; and Dr. A. N. Bell, of N. Y., Secretary.

### REPORT ON DISINFECTANTS.

The first paper called up was the Report on Disinfectants, which was read by Dr. E. M. Hunt, of New Jersey, chairman of the committee. On motion to refer it to the Committee on Publication, it was brought under discussion by Dr. T. Antisell, who, though he considered the report in many respects an able one, did not regard it sufficiently elaborate, particularly in the special utility of dry heat for the destruction of fungous organisms resulting from the exposed excreta of persons sick of the cholera. While he regarded the most scrupulous cleanliness and care in the removal as speedily as possible, of all accumulations of whatever kind from such persons; yet he thought that here disinfectants had their chief application, and that if means could be devised for the thorough application of heat to all such accumulations, the poisonous quality for the diffusion of the epidemic would be destroyed. Of ozone and other disinfectants dwelt upon by the committee, he thought them of much less importance, and that they occupied too large a space in the report, to the exclusion of that which he considered so much more useful.

Drs. Green, C. A. Lee, E. R. Squibb and Jas. Hibberd took issue with Dr. Antisell as to the province of the committee in the report, regarding it in the main as sufficiently elaborate, and more strictly in accordance with what reports for the Transactions of the Association should be, than if it went into collateral subjects.

Drs. Hunt and Bell defended the report on the ground that it was necessarily a chief effort on the part of the committee to present a resume of such facts as had from time to time been brought to notice in the history of disinfectants; and that it would not only be out of the province of the committee, but quite impossible, to report upon the special indications for the application of disinfectants to the various supposed causes of disease, such as the one presented by Dr. Antisell.

The motion to refer the paper to the Committee on Publication was then put, and the paper was so referred.

On motion, it was voted that Dr. Antisell be requested to prepare a paper for the next Session of the Association, *On the Causes of Epidemics.*

### SECOND SESSION, MAY 2.

### THE USE OF PERMANGANATE OF POTASH.

Dr. B. F. Craig read a paper on the use of permanganate of potash for the purification of water, especially during the prevalence of epidemic cholera.

The points of the paper were presented by Drs. C. A. Lee and E. R. Squibb, namely, that the disinfecting agent in the use of the permanganate of potassa and allied substances—was ozone. According to the remarks of Dr. Squibb, the power of disinfecting substances generally, excepting some, such as charcoal, which are absorbents, can be measured in a certain degree by the facility with which they give out or abstract oxygen. The paper under discussion was defective, in that the author assumed that the organic matter present in nature is the cause of cholera; and also that he took no note of the deposit of carbonate of potassa that would take place even to a mischievous degree in the use of the remedy. Yet, upon the whole, the paper should be regarded as a contribution to the important subject of disinfectants. It was, on motion, voted to commend it to the Committee on Publication.

### COMPULSORY VACCINATION.

Compulsory Vaccination was presented as a subject of discussion by Dr. A. N. Bell, chairman of the committee on that subject. While he regarded the literature of *vaccination for the profession* exhausted, he considered the field still large for the purposes of the committee, namely, the diffusion of information among the people; and his chief object in presenting the subject in this manner, instead of by formal report, was to elicit the expression of the Section as to the best means of diffusing information. In the Southern States, especially, a new field was now open for reaching a large number of persons requiring vaccination and revaccination, in order to put a stop to the ravages of small-pox, and he hoped that some of the gentlemen present would be able to assist the Committee in devising means for such a purpose.

Dr. Wm. M. Charters, of Georgia, described the terrible ravages of small-pox among the negroes at Savannah on the arrival of General Sherman's army, and attributed it, not to any want of a correct appreciation of the utility of vaccination, but to the *imperfect manner* in which vaccination was performed.

It was the practice, to a great extent, to commit this subject to unprofessional persons. In the Southern States, especially, masters and overseers thought themselves equally competent with physicians for so simple an operation. But the result justified the conclusion, that the disease they communicated was frequently a *vaccinoid* sore only, and not a vaccine vesicle. The operation was performed more with reference to making a *sore* than anything else, and that was regarded as the prophylactic, but exposure proved to the contrary. In other cases, however, and these were by no means insignificant, vaccination had been well performed and it had taken perfectly, but the susceptibility to it and to small-pox not having been exhausted, the individual was still liable. He therefore advocated exhaustive vaccination to begin with; let the operation be repeated until it ceases to produce an impression, and he believed susceptibility to small-pox would be destroyed for the whole lifetime of the individual. To accomplish this, he believed in the necessity of compulsory laws, requiring every person to be thoroughly vaccinated, under such penalties as would insure compliance.

Drs. Lee and Hibberd referred to the ineffectual State laws on the subject, and also to the no less ineffectual laws of England, and thought that something more was required.

Dr. Nebinger did not believe that such laws could ever be made effectual in this country, and thought that the work of the Committee to diffuse information—which, so far as vaccination without danger, or a belief in its prophylactic powers was concerned, was effectual—only now need be pressed to the equal necessity of *re*vaccination. On the necessity of *re*vaccination, he knew that there were yet many physicians and a multitude of other people needing information. He cited several cases in illustration. He hoped that the Committee would be continued, and that every member of the Section would do his utmost to press upon the attention of the profession and the public the facts that had been already presented in the last year's report.

Dr. Toner expected much good to result from registration, a subject that was now attracting much attention; and when it was so far advanced that correct information could be gained as to who was and who was not vaccinated, he thought that one great obstacle would be removed—the necessity of universal vaccination would be so much more apparent that the purpose of the Committee would be much facilitated.

On motion, it was voted that the Section recommend that the Committee be continued.

Dr. C. A. Lee introduced *leakage of gas pipes* as a subject well worthy of the consideration of the Section and of the Association.

On motion, it was voted that Dr. J. C. Draper, of New York, be requested to prepare a report for the next session of the Association on the leakage of gas pipes.

### THIRD SESSION, MAY 3.

#### ON ALCOHOL AND ITS RELATIONS TO MAN.

This paper, on Alcohol and its Relations to Man, by Dr. Gerard F. Morgan, of Maryland, was presented and in part read by Dr. Dunbar, member of the special Committee with Dr. Morgan to present a report upon this subject.

After some discussion, it was, on motion, voted, that the report presented by the Committee on the subject be referred to a new committee, to report at the next session.

Dr. Dunbar, of Maryland, Dr. T. Antisell, D. C., and Dr. N. S. Davis, Ill., were nominated, and the request referred to the Association.

Adjourned *sine die.*

